# Genetic Interactions Between *BOB1* and Multiple 26S Proteasome Subunits Suggest a Role for Proteostasis in Regulating *Arabidopsis* Development

**DOI:** 10.1534/g3.118.300496

**Published:** 2018-02-27

**Authors:** Elan W. Silverblatt-Buser, Melissa A. Frick, Christina Rabeler, Nicholas J. Kaplinsky

**Affiliations:** Department of Biology, Swarthmore College, Swarthmore, PA 19081

**Keywords:** BOB1, NudC, proteasome, plant development, proteostasis, AS1, AS2, FIL

## Abstract

Protein folding and degradation are both required for protein quality control, an essential cellular activity that underlies normal growth and development. We investigated how *BOB1*, an *Arabidopsis thaliana* small heat shock protein, maintains normal plant development. *bob1* mutants exhibit organ polarity defects and have expanded domains of KNOX gene expression. Some of these phenotypes are ecotype specific suggesting that other genes function to modify them. Using a genetic approach we identified an interaction between *BOB1* and *FIL*, a gene required for abaxial organ identity. We also performed an EMS enhancer screen using the *bob1-3* allele to identify pathways that are sensitized by a loss of *BOB1* function. This screen identified genetic, but not physical, interactions between *BOB1* and the proteasome subunit *RPT2a*. Two other proteasome subunits, *RPN1a* and *RPN8a*, also interact genetically with *BOB1*. Both *BOB1* and the *BOB1*-interacting proteasome subunits had previously been shown to interact genetically with the transcriptional enhancers *AS1* and *AS2*, genes known to regulate both organ polarity and KNOX gene expression. Our results suggest a model in which *BOB1* mediated protein folding and proteasome mediated protein degradation form a functional proteostasis module required for ensuring normal plant development.

Protein homeostasis (proteostasis) is a fundamental prerequisite for cellular function and, by extension, for growth and development in multicellular organisms. Proteostasis is established and maintained through the interplay of two core cellular processes, protein folding and protein degradation. Co- and post-translational protein folding are facilitated by protein chaperones while most regulated protein degradation is performed by the 26S proteasome (26SP). Protein chaperones are a diverse group of proteins, many are encoded by evolutionarily conserved heat shock protein (HSP) genes. The activity and the functional importance of both HSP chaperones and the 26SP are well established in plants and include a wide range of roles in plant growth and development (Ishiguro *et al.* 2002; Queitsch *et al.* 2002; Ueda 2004; Jenik and Barton 2005; Perez *et al.* 2009; Wang *et al.* 2009; Ueda *et al.* 2011). Both processes have been extensively studied but the importance of interactions between HSP mediated protein folding and protein degradation by the 26SP has not been characterized in much detail.

Protein misfolding is predicted to occur at appreciable rates (2–9% of all cellular proteins) even under normal conditions (Drummond and Wilke 2008). In the absence of sufficient chaperone or proteasome activity, misfolded proteins accumulate in the cytoplasm and can form cytotoxic aggregates. The first line of defense against the formation of these aggregates is the small HSPs (sHSPs). sHSPs are protein chaperones that bind to and prevent the irreversible aggregation of misfolded proteins in an ATP independent manner (Basha *et al.* 2012). As their name suggests, sHSPs are small proteins (<40 kD). They contain an alpha-crystallin domain (ACD), have the ability to inhibit protein aggregation *in vitro*, and localize to cytoplasmic heat shock granules in heat stressed plant cells (Haslbeck *et al.* 2005; Siddique *et al.* 2008; Wallace *et al.* 2015). BOB1 is a non-canonical Arabidopsis sHSP that exhibits all of these characteristics. It is required for organismal thermotolerance and contains a NudC domain that is predicted to have structural homology with ACD-containing sHSPs (Garcia-Ranea *et al.* 2002; Perez *et al.* 2009). As is true for BOB1, A. *nidulans*, *C. elegans*, and human NudC proteins have also been shown to have *in vitro* chaperone activity using model substrates (Chiu *et al.* 1997; Dawe *et al.* 2001; Faircloth *et al.* 2009; Zheng *et al.* 2011). *In vivo*, human NudC proteins function as Hsp90 co-chaperones and their client proteins have been systematically identified (Taipale *et al.* 2014). It is, of course, possible that NudC proteins could have additional uncharacterized functions in addition to their demonstrated chaperone activity. *BOB1* is an essential gene in Arabidopsis and NudC loss of function mutations are also lethal in *A. nidulans*, *C. elegans* and Drosophila.

Analysis of the arrested globular embryos of null alleles of *BOB1* (*bob1-1* and *bob1-2*) revealed early and severe developmental defects including an expanded apical meristem and associated *STM* expression. *STM*, a KNOX gene essential for shoot meristem function, is normally expressed only in the central domain of the apical half of the Arabidopsis embryo. In *bob1* null mutants STM expression expands into the lateral apical domains of the embryo. The expansion of the meristem is accompanied by a lack of cotyledon development and an associated loss of expression of genes normally expressed in lateral organs (Jurkuta *et al.* 2009). These results demonstrate that *BOB1* negatively regulates KNOX gene expression.

*BOB1* is also required for post-embryonic development. The hypomorphic *bob1-3* allele exhibits pleiotropic developmental defects. *bob1-3* plants have short roots, small serrated leaves, short branched inflorescences, and inflorescence and floral meristem defects that result in pin-formed meristems and floral organ number defects (Perez *et al.* 2009). Many of these phenotypes are reminiscent of mutants defective in auxin signaling or transport. The serrations on the margins of *bob1-3* leaves are dependent on *PIN1* activity, supporting the idea that *BOB1* is required for auxin mediated developmental patterning (Jurkuta *et al.* 2009; Kaplinsky 2009). However, the molecular mechanisms by which this sHSP affects plant development are not self-evident from any of these studies.

*BOB1* interacts genetically with both *AS1* and *AS2*, providing a clue about a developmental pathway that requires *BOB1* activity. *AS1* and *AS2* are transcriptional regulators that play roles in establishing meristem boundaries by repressing KNOX expression as well as reinforcing ab-adaxial polarity during leaf development (Iwakawa *et al.* 2002; Iwasaki *et al.* 2013; Machida *et al.* 2015). An allele of *BOB1* called *eal-1* was identified in an *as1* enhancer screen (Ishibashi *et al.* 2012). Surprisingly, *eal-1* has the same mutation as the *bob1-3* (G141E) allele. This is the only viable allele of *BOB1* with known phenotypes (Perez *et al.* 2009). *eal-1*; *as1* and *as2* double mutants have abaxialized filamentous leaves and exhibit increased levels of KNOX and *ETT* expression in their shoot apices. *ETT* functions to enhance abaxial identity and is a direct target of the AS1-AS2 complex (Iwasaki *et al.* 2013). The polarity defects in *eal-1*; *as2* plants were suppressed in an *ett* background suggesting that *ETT* is downstream of both *BOB1* and *AS1* and *AS2* (Ishibashi *et al.* 2012).

The aim of this study was to identify *BOB1* dependent developmental pathways in order to understand the requirement for this sHSP in ensuring normal development. We used a genetic approach, reasoning that *bob1-3* enhancers would be caused by mutations in genes and pathways that are sensitive to reductions in *BOB1* activity.

## Materials and Methods

### Plant stocks and growth conditions

*bob1-3* and *bob1-1* were both back crossed into Ler background six times before being used to analyze TH phenotypes (both alleles) and for the EMS mutagenesis (*bob1-3*). TH plants in both Col-O and Ler backgrounds were generated by crossing *bob1-3* homozygotes to *bob1-1* heterozygotes. Seeds produced by these crosses segregate *bob1-3/bob1-1* (TH phenotype) and *bob1-3/+* (WT phenotype) plants in a 1:1 ratio. *fil* mutants and proteasome subunit T-DNA insertion lines were obtained from the ABRC (File S1). Plants were grown on soil under standard long-day greenhouse conditions with supplemental lighting. Plants grown on plates were grown on 0.5x Murashige and Skoog (0.5x MS) media containing 1% sucrose at 22° under constant light conditions in E-30B growth chambers (Percival Scientific).

### EMS mutagenesis

5000 *bob1-3* seeds in a Ler background were soaked in a 0.2% EMS solution on a rocker for 12 hr. They were then rinsed eight times with water and planted on soil. M_1_ Seeds were collected from pools of 4-5 M_0_ self-fertilized plants.

### Leaf shape measurements

The fifth and sixth leaves from three week old plants were flattened, taped to white paper with transparent packing tape, and scanned at 600dpi using a Canon iR-ADV C5530. The scanned images were used to measure the length and mid-length width of each leaf lamina as well as the depth of the deepest serration on each leaf using ImageJ (Schneider *et al.* 2012).

### BOM1 mapping and cloning

DNA from 538 mutant individuals in a *bom1* mapping population was prepared using a DNeasy Plant Maxi Kit (Qiagen). A NEBNext DNA library prep set for Illumina (NEB) was used to prepare the sequencing library that was sequenced using an Illumina HiSeq (Illumina). Reads were mapped to the TAIR9 reference genome using SHORE and *bom1* was mapped using SHOREmap (Schneeberger *et al.* 2009).

### RT-PCR

RNA from five day old seedlings grown in 0.5x MS liquid media was prepared using RNeasy plant mini kits (Qiagen). It was quantified using a Nanodrop ND-1000 (Thermo Scientific) and reverse transcribed using M-MuLV reverse transcriptase (NEB). The following primers were used for PCR reactions with a 55° annealing temperature and 35 cycles: ACTIN-F, 5′-GAAGAACTATGAATTACCCGATGGGC-3′; ACTIN-R, 5′-CCCGGGTTAGAAACATTTTCTGTGAACG-3′; RPT2a-F, 5′- CACCATGGGACAAGGACCATC-3′, RPT2a-R 5′- TTACATGTAGAGGCCTTCAG-3′.

### MG132 treatment

MG132 (Cayman Chemical) was resuspended at 100 mM in DMSO before being added to MS agar (1%) media. Col-O, *bob1-3*, *bom*, *rpt2a-2*, and *bob1-3*; *bom* seeds were plated and stratified at 4° for two days. The plates were then transferred to a 22° incubator in a vertical orientation, exposed to light for five hours, and then wrapped in two layers of aluminum foil. After five days of growth the plates were scanned using an Expression 1600 scanner (Epson) and hypocotyl lengths were measured using ImageJ (Schneider *et al.* 2012).

### BiFC

Full length *RPT2a* and *BOB1* cDNA clones were amplified from an *Arabidopsis thaliana* Col-O cDNA pool using two sets of primers. A common forward primer was used while one reverse primer for each gene included the stop codon (for C-terminal fusions) while the other did not (used for N-terminal fusions). The following primers were used: RPT2a-F 5′CACCATGGGAGAAGGACCATC-‐3′, RPT2a-R-STOP 5′-TTACATGTAGAGGCCTTCAC‐3′, RPT2a-R-NOSTOP 5′-CATGTAGAGGCCTTCAGGGA‐3′, BOB1-F 5′ CACCATGGCGATTATCTCTGAGGTAGAAG-‐3′, BOB1-R-STOP 5′- TCAGTTAAACTTTGCATTTGAGAAGTCCAT‐3′, BOB1-R-NOSTOP 5′- GTTAAACTTTGCATTTGAGAAGTCCAT‐3′. Amplified cDNAs were cloned into pENTR/D‐TOPO (Invitrogen) and subsequently recombined into pNYFP‐x and pCCFP‐x, or x-pNYFP and x-CCFP (Kim *et al.* 2009), respectively, to generate N-terminal and C-terminal fusions to both fluorescent protein fragments.

*Agrobacterium tumefaciens* strain GV3101 was transformed with each construct by electroporation. Transformants were grown for 2 days at 28° shaking at 250 rpm. Cells were collected by centrifugation and resuspended in 1 mL water and the OD_600_ of these cultures was adjusted to 0.2. Cultures were combined so that every RPT2a/BOB1 and NYFP/CCFP pairing was created as well as other combinations including positive controls known to interact in BiFC assays (CCFP-BOB1/NYFP-BOB1; BOB1-CCFP/NYFP-BOB1; 14-3-3-NYFP/14-3-3-CCFP) and negative controls previously shown not to interact in BiFC assays (BOB1-CCFP/BOB1-NYFP; CCFP-BOB1/BOB1-NYFP). Combined cultures were pelleted and resuspended in 1 mL of induction media (10 mM MgCl2; 10 mM MES, pH 5.6; 150 mM acetosyringone). Induced cultures were infiltrated into three to four week old *Nicotiana benthamiana* leaves using a syringe. Treated leaf tissue was visualized using an SP5 AOBS confocal microscope (Leica) 48 hr after infiltration.

### Scanning electron microscopy

Fresh tissue was imaged using low vacuum mode on a Quanta 200 scanning electron microscope (FEI) equipped with a cooled stage.

### Data and reagent availability

Strains described in this paper are available upon request. The sequence reads generated for this project have been deposited at the SRA under BioSample accession SAMN07416858.

## Results

### BOB1 phenotypes are different in Ler and Col-O genetic backgrounds

**Figure 1:**
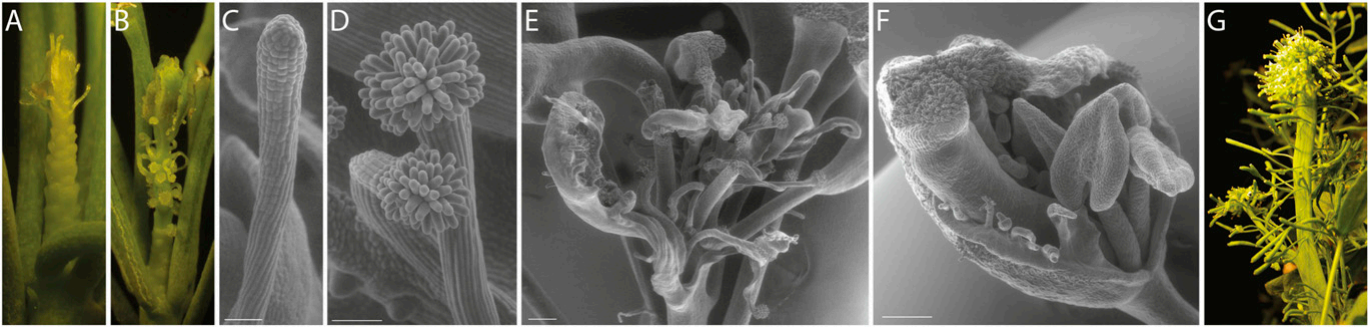
Floral and inflorescence phenotypes of *bob1-3/bob1-1* Ler plants. *bob1-3/bob1-1* plants in a Ler background produce meristems that cease to produce normal flowers, instead producing radialized organs (A, B). These filamentous lateral organs are either bare (A, C) or topped by stigmatic papillae (B, D). Inflorescences also terminate in mixtures of abnormal floral organs (E). Intermediate flowers exhibit abaxialized features including exposed ovules (F). TH plants also often develop with fasciated stems (G). Scale bars are 50μm (C,D) and 250μm (E,F).

*bob1-3* phenotypes include small plants with short roots, serrated leaf margins, and abnormal floral organ numbers (Perez *et al.* 2009). These phenotypes are very similar in Col-O and Ler ecotypes. By reducing the dosage of *BOB1* we uncovered other ecotype specific phenotypic differences. We combined the *bob1-1* null allele and the *bob1-3* partial loss of function allele to create *bob1-1/bob1-3* trans-heterozygotes (THs). In a Ler background these plants had inflorescence phenotypes that were markedly different from the pin-formed meristems that develop in *bob1* TH plants in a Col-O background (Perez *et al.* 2009). Several types of lateral organs are produced by Ler TH inflorescences, often on the same plant. Most Ler TH inflorescence meristems produce a series of relatively normal flowers followed by increasingly abnormal flowers and then finally filamentous structures and arrested primordia. Based on their placement on the flank of the inflorescence meristem these filamentous structures appear to be derived from flowers. On some plants the filaments are bare (Figure 1 A, C) while on other plants they terminate in stigmatic papillae, consistent with a floral derivation (Figure 1B, D). A second class of Ler TH inflorescences terminates in a mixture of structures including isolated carpels, leaf like structures with ectopic ovules on their margins, and filamentous organs (Figure 1E). Intermediate flowers (between the relatively normal early flowers and the terminated meristems) exhibit severe polarity defects including visible external ovules (Figure 1F). Finally, Ler TH plants can occasionally develop fasciated meristems with strap like stems (Figure 1G) suggesting that, in addition to the patterning phenotypes described above, at low *BOB1* dosages control over meristem size is also lost.

*bob1* THs in a Col-O background are completely sterile and do not produce any seeds. In contrast, the relatively normal early Ler TH flowers are fertile and set seed. The abnormal infloresences observed in Ler THs are qualitatively different from what we observed in a Col-O background. These differences suggest the existence of genetic modifiers that affect *bob1* phenotypes. We reasoned that identifying these modifiers and other genes that enhance *bob1* phenotypes might provide insights into *BOB1*’s developmental functions.

### bob1-3 enhances fil phenotypes

Homozygous *bob1-3* mutants never exhibit the filamentous flower phenotype we see in *bob1-3/bob1-1* THs. This phenotype, which we only observe in a Ler background, is similar to the filament-like structures described in *filamentous flower* (*fil*) mutants. *fil* mutant plants, like *bob1* THs, also produce a number of relatively normal flowers before terminating in clusters of filamentous organs. *FIL* encodes a *YABBY* gene required for floral meristem identity, flower formation, and flower development (Chen *et al.* 1999) and has a demonstrated role in establishing ab-adaxial polarity in developing leaves. As is true for *BOB1*, *FIL* also functions as a negative regulator of KNOX gene expression (Kumaran *et al.* 2002; Jurkuta *et al.* 2009; Ishibashi *et al.* 2012; Iwasaki *et al.* 2013). These similarities suggest a functional overlap between *BOB1* and *FIL*.

**Figure 2:**
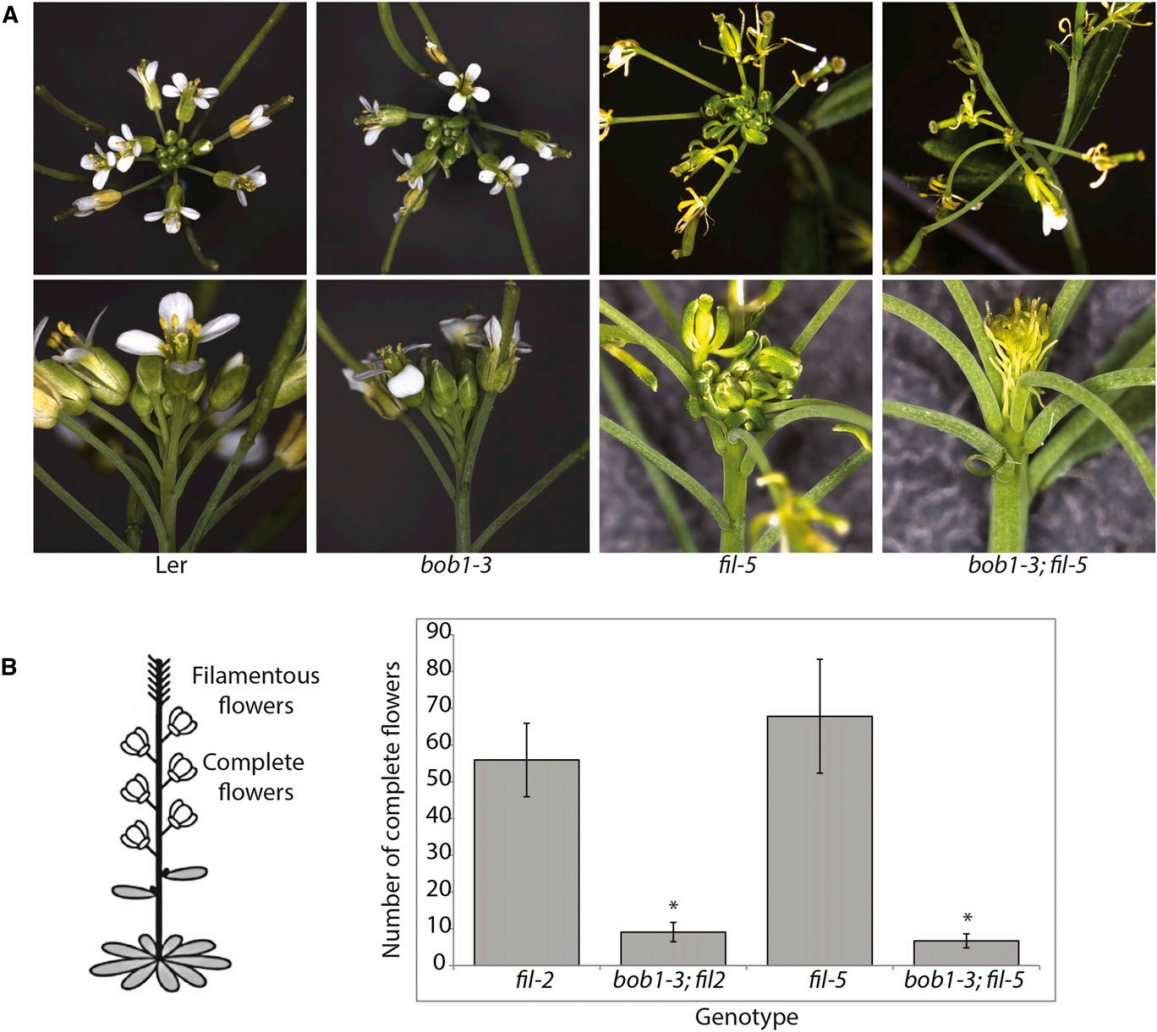
Floral and inflorescence phenotypes in Ler, *bob1-3*, *fil-5*, and *bob1-3*; *fil-5* plants. Top and side views of Ler, *bob1-3*, *fil-5*, and *bob1-3*; *fil-5* inflorescences (A). Quantitation of the number of complete flowers produced before the onset of filamentous flowers (B). * indicates a significant difference from the respective *fil* single mutant (2-tailed *t*-test *P* < 0.005). Diagram adapted from Chen *et al.* (1999).

To investigate whether *bob1-3* mutants would enhance the *filamentous flower* phenotype we crossed it with intermediate (*fil*-2) and strong (*fil*-5) *fil* alleles in a Ler background. The petals of *bob1-3* flowers are morphologically normal although *bob1-3* flowers often have more than four petals per flower (Perez *et al.* 2009). In contrast, *fil-5* mutant flowers have small twisted petals (Figure 2A). Wild type and *bob1-3* plants never produce filamentous flowers. *fil-2* and *fil-5* plants that are wild type or heterozygous for *bob1-3* produce more than 50 normal flowers before producing filamentous organs. *fil-2* and *fil-5* plants homozygous for *bob1-3* never produced more than 12 normal flowers before making filamentous organs. In these double mutants, the small number of early flowers produced look like *fil* flowers while all later flowers are converted into filamentous organs (Figure 2A, B). This phenotype suggests that the filamentous organ phenotypes seen in *bob1* THs may be due to disruptions of a developmental pathway that involves YABBY mediated establishment of organ polarity.

### bom1 is a bob1-3 enhancer

In addition to investigating genetic interactions between *BOB1* and *FIL* we also undertook a non-targeted approach to discover genes that interact genetically with *BOB1*. Since Ler THs are fertile we decided to screen for modifiers in a Ler background. We used ethylmethanesulfonate (EMS) to mutagenize *bob1-3* plants. M_1_ plants were planted, allowed to self-fertilize, and M_2_ progeny were screened for phenotypes similar to those observed in *bob1-3/bob1-1* TH plants. We screened for multiple phenotypes including reduced plant size, defects in floral organ polarity, fasciated stems, and abaxial leaf spurs. Putative mutants were crossed to wild type plants, the F_1_ progeny were self-fertilized, and F_2_ plants were grown out and genotyped to verify that their phenotypes were *bob1-3* dependent and not expressed in wild type plants. We screened a total of 414 M_2_ pools and identified five *bob1-3* dependent mutants, one of which is described here.

*bobber modifier* (*bom1*) was identified as a *bob1-3* modifier that enhances several *bob1-3* phenotypes in a manner resembling *bob1* TH phenotypes. These include small plant size and heavily serrated, narrow leaves. *bom1* behaves as a single recessive mutation in a *bob1-3* background. We backcrossed *bom1* to *bob1-3* in Col-O and wild type Col-O plants six times before phenotypic analysis to remove unlinked EMS induced mutations. All further analyses described in this paper were performed in a backcrossed Col-O background.

**Figure 3:**
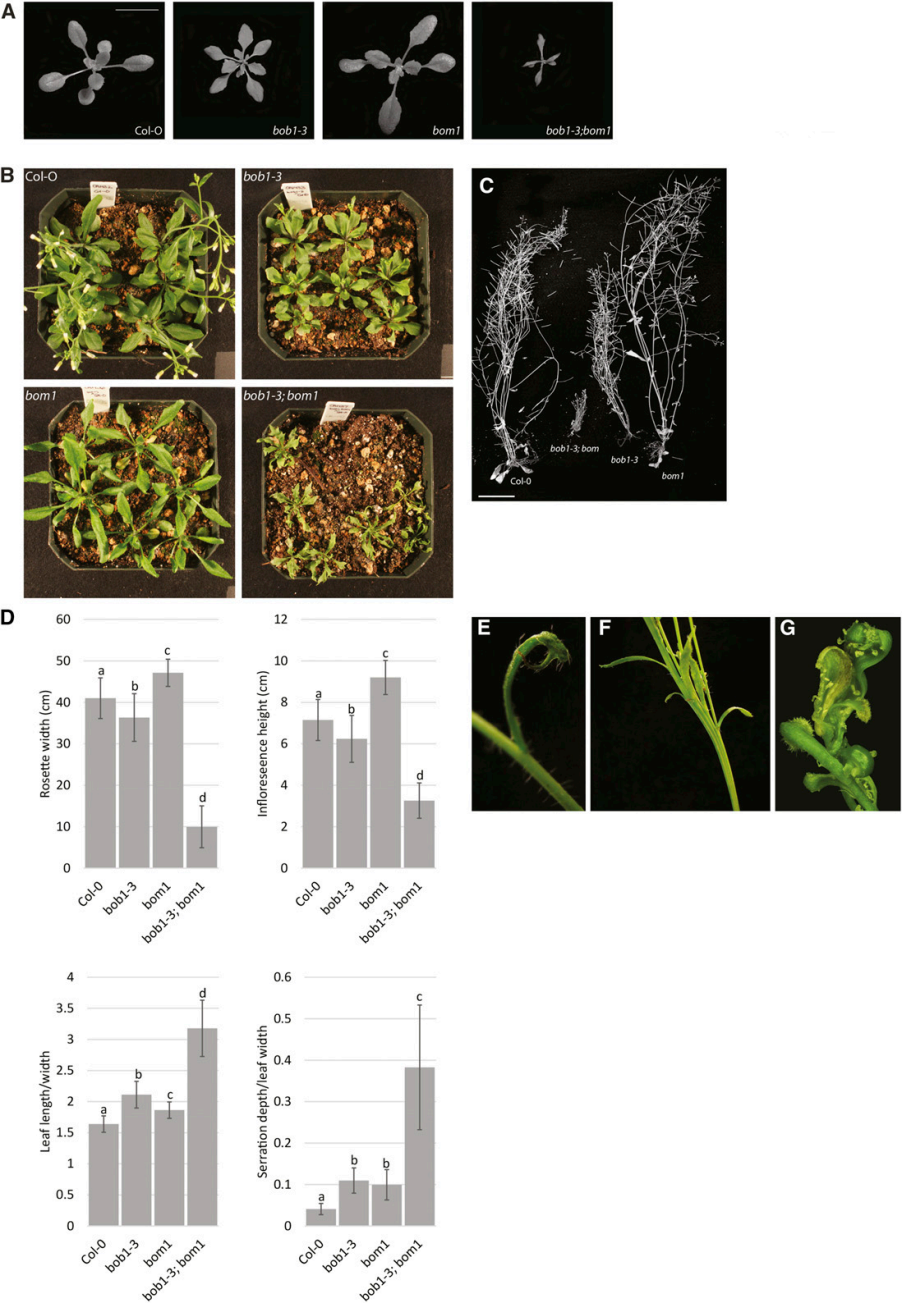
*bom1* developmental phenotypes. Size and leaf serration phenotypes in three (A) and five (B) week old plants. Height differences in mature, dried plants (C). Rosette widths and inflorescence heights were measured in four week old and mature plants, respectively. Leaf lamina length/width and serration/leaf width ratios were measured for leaves five and six in three week old plants. Letters indicate significant differences among genotypes (Bonferroni corrected 2-tailed unpaired *t*-tests, *P* < 0.05) (D). *bob1-3*; *bom1* phenotypes include abaxial leaf spurs (E), stem fasciation (F), and abaxialized flowers with visible external ectopic ovules (G). Scale bars are 1cm in A and 2cm in C. Error bars are +/− SD.

Both *bob1-3* and *bom1* single mutants have serrated and narrow leaves. *bob1-3* mutant plants have smaller rosette diameters and shorter inflorescences compared to wild type plants (Perez *et al.* 2009). This is in contrast to *bom1* mutant plants whose rosette diameters and shoot heights are larger than those of Col-O plants. *bob1-3*; *bom1* leaves are narrower and more serrated than those of either single mutant and the rosette diameter and shoot height of double mutant plants are smaller than those of Col-O, *bob1-3*, or *bom1* plants (Figure 3A-D). These epistatic genetic interactions suggest that *BOB1* and *BOM1* function in a shared biological pathway (Roth *et al.* 2009).

Similar to *bob1* THs, *bob1-3*; *bom1* double mutants also have abaxial leaf spurs, fasciated stems, and their inflorescences terminate in clusters of abaxialized flowers. These flowers lack obvious sepals, petals, or stamens. The carpeloid organs that are produced at inflorescence termini have exposed ovules on their margins (Figure 3E-G). We occasionally observe tiny leaf spurs in *bob1-3* in a Col-O background but they are never as pronounced as those seen in *bob1-3*; *bom1* plants.

### bom1 is an allele of RPT2a

**Figure 4:**
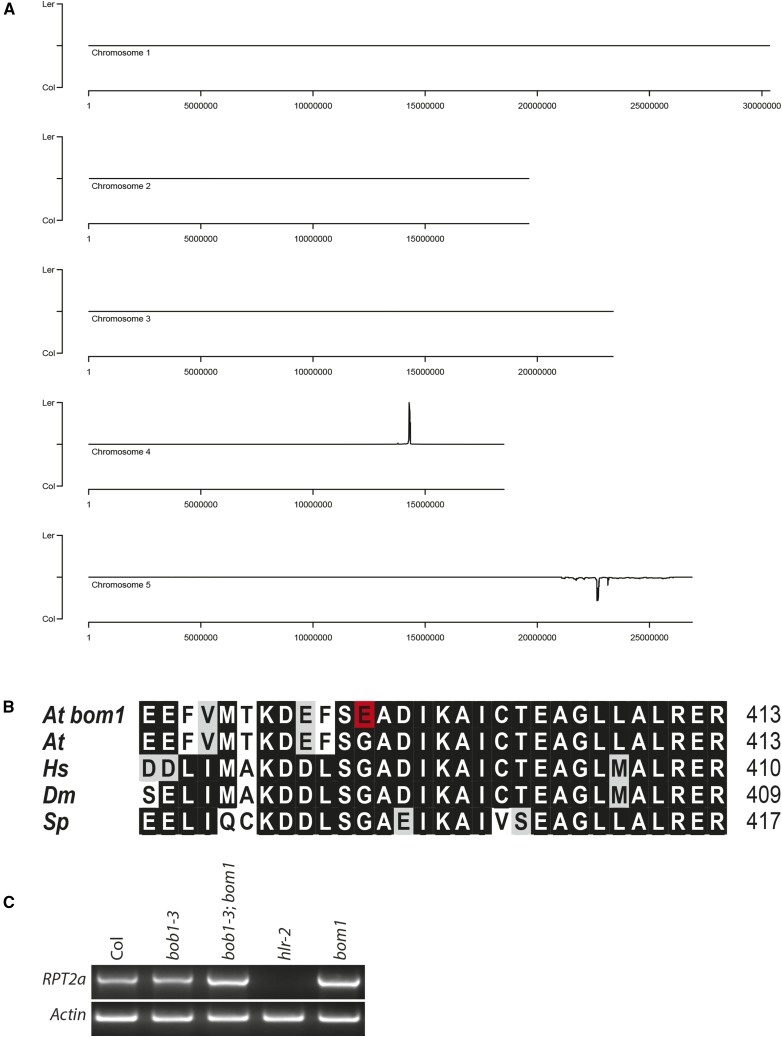
Cloning and characterization of *bom1*. *BOM1* was simultaneously mapped and cloned using a NGS approach. *bom1* and *bob1-3* were generated in Ler and Col-O backgrounds, respectively, and their positions can be seen as peaks of enrichment in Ler and Col SNPs (A). A protein lineup of BOM1/RPT2a. The G395E mutation in *bom1* is highlighted in red (B). RT-PCR was used to amplify full length *RPT2a* transcripts from mRNA isolated from the indicated genotypes (C).

We simultaneously mapped and cloned *BOM1* using a next generation sequencing approach (Schneeberger *et al.* 2009). *bob1-3*; *bom1* plants in a Ler background (in which the mutation was generated) were crossed to *bob1-3* plants in a Col-O background. The F_1_ progeny were self-fertilized and the resulting F_2_ plants segregated the *bob1-3*; *bom1* phenotype in a *bob1-3* background. DNA was extracted from 548 *bob1-3*; *bom1* plants, pooled, and sequenced to 82x coverage. The SHORE and SHOREmap software packages (Schneeberger *et al.* 2009) were used to identify Col/Ler SNP allele frequencies across the entire Arabidopsis genome. Two regions of enrichment were identified. As expected, a region of enrichment of Col SNP alleles in the *BOB1* region of chromosome 5 is consistent with the Col origin of the *bob1-3* allele. Enrichment of Ler SNPs was observed only on chromosome 4 with a peak at 14.3Mb (Figure 4A). We decided to focus on G→A /C→T mutations (characteristic of EMS mutagenesis) that resulted in non-synonymous changes as candidate mutations. The closest non-synonymous G→A mutation to the mapping peak was a mutation that results in a G395E amino acid change in AT4G29040. AT4G29040 encodes the *RPT2a* subunit of the 19S regulatory particle of the 26S proteasome. The mutation in the *bom1* allele is located close to the AAA-ATPase domain of *RPT2a* and affects a glycine residue that is invariant among plants (Arabidopsis), animals (humans and flies), and fungi (*Sacchromyces pombe*) (Figure 4B).

In order to confirm that *bob1-3*; *bom1* phenotypes are caused by a mutation in *RPT2a* we performed a complementation test using *rpt2a-2* (SALK_005596), a null allele of *RPT2a* that was first described as *halted root-2* (*hlr-2*) (Ueda 2004). *rpt2a-2* was crossed to a *bob1-3*; *bom1/+* plant. F_1_ plants were genotyped and a *bob1-3/+*; *rpt2a-2/bom1* plant was self-fertilized. If *bom1* is an allele of *RPT2a* we would expect 1/4 of the F_2_ plants to exhibit the double mutant phenotypes while if *bom1* is not an allele of *RPT2a* we would expect 1/16 of the F_2_ plants to exhibit the double mutant phenotypes. 12 out of 43 plants in the F_2_ population had *bob1-3*; *bom1* double-mutant phenotypes. These plants were genotyped for *bob1-3*, *bom1*, and *rpt2a-2*. As expected, all plants were homozygous for *bob1-3* and we identified *bom1* and *rpt2a-2* homozygotes as well as *bom1/rpt2a-2* plants among the plants with double mutant phenotypes. This lack of complementation shows that the *bom1* enhancement of *bob1-3* phenotypes is caused by a mutation in *RPT2a*. The increased size of *bom1* plants (Figure 3) is also consistent with reports that *rpt2a* mutants are larger than wild type plants (Kurepa *et al.* 2009; Lee *et al.* 2011).

Three other *RPT2a* alleles have been described in addition to *bom1* and *rpt2a-2*. *rpt2a-1* is the original *halted root-1* (*hlr-1*) allele identified in a Wassilewskija background and has a 13bp deletion in its first intron (Ueda 2004). Three T-DNA insertional alleles, *rpt2a-2* (*hlr-2*), *rpt2a-3*, and *rpt2a-4* are all in a Col-O background. No full length RNA transcripts accumulate in any of these T-DNA alleles making it likely that they are all loss of function alleles (Lee *et al.* 2011). We propose that *bom1* be designated as *rpt2a-5*.

The phenotypes of the *rpt2a-2*; *bob1-3* and *bom1*; *bob1-3* double mutants generated during the complementation test were very similar. This suggests that the G395E mutation is a strong allele with phenotypic effects similar to the T-DNA insertional alleles. To characterize the *bom1* allele of *RPT2a* further we investigated whether *RPT2a* RNA accumulates in *bom1* mutants. *rpt2a-2* is an RNA null and, as expected, there was no detectable *RPT2a* RNA in *rpt2a-2* mutants. Full length *RPT2a* RNA was detectable in *bom1* single mutants and in *bob1-3*; *bom1* double mutants at levels similar to wild type and *bob1-3* plants (Figure 4C).

### bom exhibits MG132 hyposensitivity

**Figure 5:**
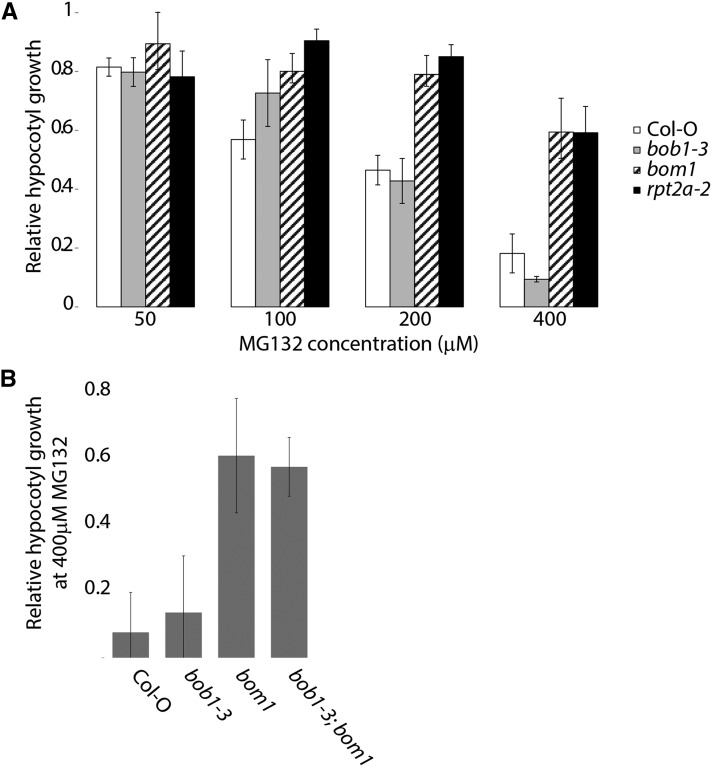
Inhibition of hypocotyl growth by MG132. The growth of etiolated seedling hypocotyls exposed to MG132 concentrations between 0-400 μM was measured and normalized to the untreated hypocotyl growth rate for each genotype (A). Normalized hypocotyl growth for single and double mutants exposed to 400 μM MG132 (B). Error bars are +/− SD.

The *rpt2a2* null mutant as well as mutations in other proteasome regulatory particle subunits such as *rpn10* and *rps12* are, paradoxically, less sensitive to the proteasome inhibitor MG132 than wild type plants (Kurepa *et al.* 2008). To determine if *bom1* shares this phenotype we measured hypocotyl elongation in MG132 treated etiolated seedlings. *bom1* and *rpt2a-2* mutants both exhibited increased MG132 tolerance compared to either Col-O or *bob1-3* plants (Figure 5A). This suggests that the G395E point mutation in *bom1* results in a similar defect as the *rpt2a-2* null allele. To investigate whether a loss of *BOB1* would affect the MG132 hyposensitivity observed in *bom1* we also grew *bob1-3*; *bom1* double mutants at 400 μM MG132. At this concentration we observe a nearly complete inhibition of hypocotyl elongation in Col-O and *bob1-3* plants. The *bob1-3*; *bom1* double mutants and *bom1* single mutants exhibited similar levels of MG132 tolerance, demonstrating that a loss of *BOB1* function does not affect this aspect of *rpt2a* mutant phenotypes (Figure 5B).

### BOB1 interacts genetically with multiple proteasome subunits

**Figure 6:**
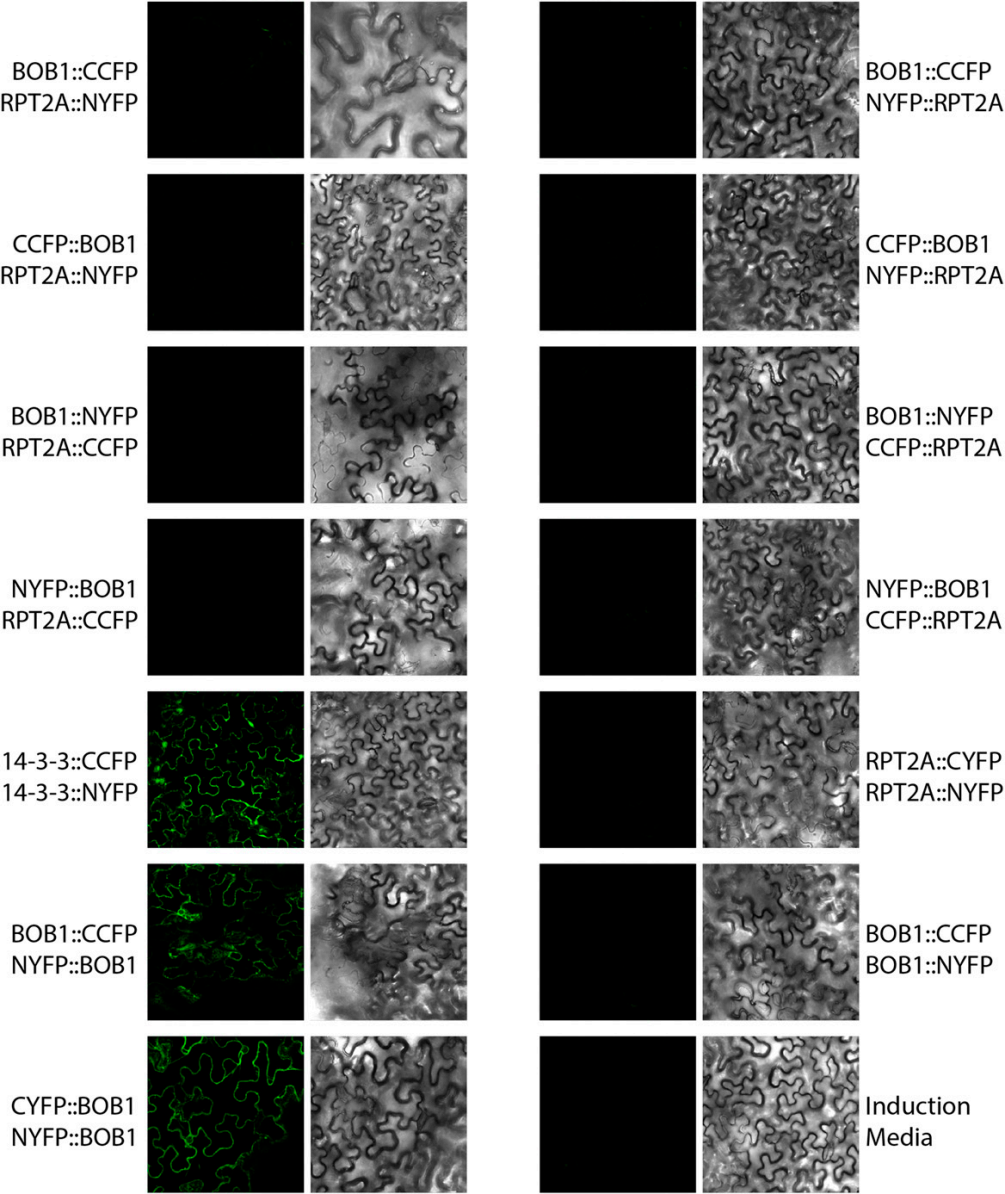
Bimolecular fluorescence complementation between BOB1 and BOM1. *BOB1* and *BOM1* BiFC constructs were tested against each other in all possible orientations. Positive controls include homo-dimerization of a 14-3-3 protein (Kim *et al.* 2009) and of BOB1 in two orientations.

To determine if a physical interaction underlies the genetic interaction between *BOB1* and *BOM1* we cloned cDNAs of both genes into BiFC vectors and tested their interaction using transient tobacco leaf transformation. The basic functional unit for sHSPs is a dimer that in turn can be incorporated into higher order structures (Basha *et al.* 2012). Consistent with this, BOB1 homodimerization can be detected using BiFC in two different combinations of BiFC constructs, (BOB1::CCFP & NYFP::BOB1, CYFP::BOB1 & NYFP::BOB1). In contrast, there was no evidence of a physical interaction between BOB1 and BOM1 in any of the BiFC construct orientations (Figure 6). It is possible that this result is a false negative and it is not possible to detect interactions between BOB1 and RPT2a using BiFC. However, based on the genetic interactions between *BOB1* and several other proteasome subunits (see below), we suspect that the genetic interactions between *BOB1* and the proteasome are not mediated by direct physical interactions.

**Figure 7:**
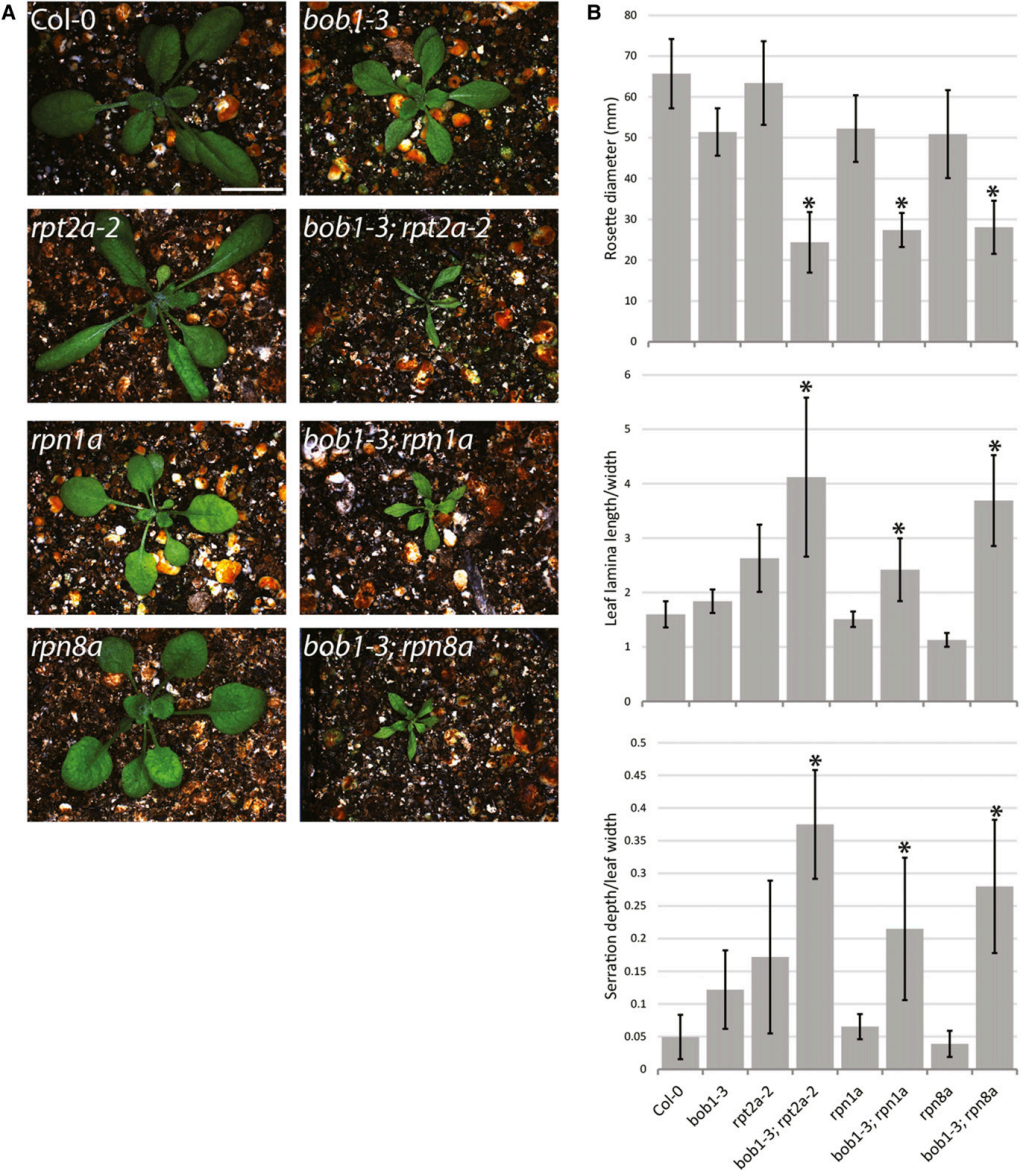
Plant growth phenotypes of *bob1-3*; 26SP double mutants. Three week old plants (A). Rosette widths were measured in four week old plants. Leaf lamina length/width and the serration/leaf width ratios of serrated leaves were measured for leaves five and six in three week old plants (B). * indicates significant differences between each double mutant and the corresponding single mutants as well as Col-0 plants (One-way ANOVA with post-hoc Tukey HSD Test, *P* < 0.05). Error bars are +/− SD. The scale bar in A is 1cm.

An alternative explanation for the genetic interaction between *BOB1* and *BOM1* is a more general interaction between *BOB1* mediated protein folding and 26SP mediated protein degradation. To test whether the interaction is specific to *BOM1* or whether it is a more general genetic interaction we made crosses between *bob1-3* plants and T-DNA insertions in multiple proteasome subunits (File S1). As expected, *bob1-3*; *rpt2a-2* double mutants exhibit a strong synergistic phenotype. In this experiment *rpt2a-2* single mutants were not significantly larger than wild type Col-O plants as has been previously reported (Kurepa *et al.* 2009) and as we observed for *bom1* (Figure 7). This discrepancy could be due to variability in our greenhouse growth conditions or the altered stress tolerance levels observed in proteasome mutants (Kurepa *et al.* 2008; Wang *et al.* 2009). We did not follow up on this observation.

In addition to *RPT2a*, T-DNA insertions in *RPN1a* and *RPN8a* exhibited strong enhancement of *bob1-3* phenotypes. Double mutants between *bob1-3* and all of these proteasome mutants were smaller than either corresponding single mutant or wild type plants. Double mutant leaves were also narrower than either single mutant. They often lacked any serrations on their margins (73% of *bob1-3*; *rpt2a*-2 leaves, 26% of *bob1-3*; *rpn1a* leaves, and 55% of *bob1-3*; *rpn8a* leaves) but, when serrations were present, they were deeper than the serrations of single mutant or wild type leaves (Figure 7).

## Discussion

We found that mutations in the proteasome subunits *RPT2a*, *RPN1a*, and *RPN8a* enhance *bob1-3* developmental phenotypes. These interactions are interesting because *BOB1* encodes a protein chaperone that prevents the aggregation of misfolded proteins and the proteasome is responsible for regulated protein degradation. The interaction between these core cellular pathways in Arabidopsis highlights the importance of proteostasis for normal development. In addition to slow growth, *bob1*; *rpt2a* double mutants have narrow, deeply serrated leaves, abaxial leaf spurs, and flowers with disrupted polarity. The floral phenotypes of this double mutant and *bob1* TH plants are similar to those of mutants in genes that regulate ab-adaxial polarity. Consistent with this, we also uncovered a genetic interaction between *BOB1* and *FIL*, a YABBY with demonstrated roles in promoting abaxial identity (Siegfried *et al.* 1999).

**Figure 8:**
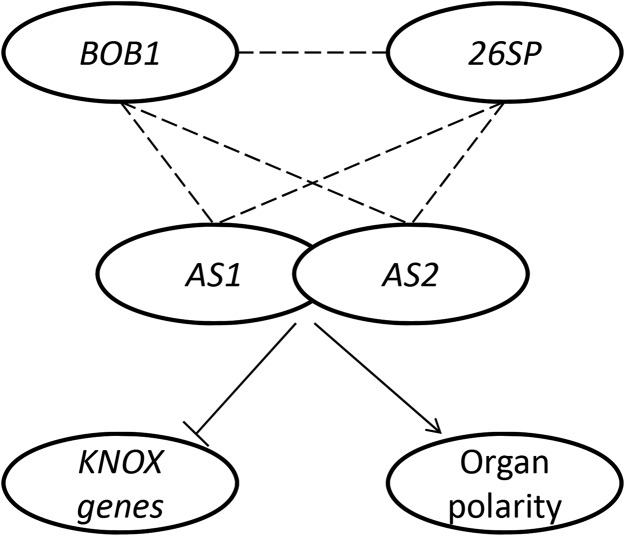
*AS1-AS2* mediated developmental events require proteostasis. Genetic interactions (dashed lines) between *BOB1* and the 26SP genetically define a proteostasis module. Both *BOB1* and the 26SP also interact genetically with *AS1* and *AS2*, known regulators of KNOX gene expression and organ polarity. This model could explain the phenotypes seen in both *BOB1* and 26SP mutants.

The interactions we have discovered define a genetic network that connects proteostasis to the *AS1-AS2* developmental pathway (Figure 8). The network consists of three sets of genetic interactions. The first is a proteostasis module defined by genetic interactions between *BOB1* and the 26SP (this work) and rests on the assumption that *BOB1*’s chaperone activities are responsible for the interactions. The second set of genetic interactions is between *BOB1* and *AS1-AS2* (Ishibashi *et al.* 2012). Finally, the third consists of genetic interactions among *RPT2a*, *RPN1a*, and *RPN8a* (the three proteasome subunits that interact genetically with *BOB1*) and *AS1-AS2* (Huang *et al.* 2006). Double mutants among these groups of genes (*BOB1*, 26SP, and *AS1-AS2*) all produce similar phenotypes and all of these genes are required for the repression of KNOX genes.

The *AS1-AS2* complex directly represses KNOX gene expression (Guo *et al.* 2008; Machida *et al.* 2015). Repression of KNOX gene expression has also been demonstrated for *RPN8a* and *RPT2a*. Mutant alleles of both of these genes exhibit abaxial leaf spurs, similar to those observed in *BOB1* THs and *bob1-3;bom1* mutants, in which KNOX genes are ectopically expressed. This shows that, as is true for *BOB1*, the 26SP can also function to negatively regulate KNOX expression (Huang and Huang 2007; Jurkuta *et al.* 2009; Ishibashi *et al.* 2012). A plausible explanation for these observations is that *BOB1*/26SP mediated proteostasis is required for normal *AS1-AS2* function (Guo *et al.* 2008; Machida *et al.* 2015).

In addition to the regulation of KNOX genes, *AS1-AS2* also functions in establishing and maintaining organ polarity in Arabidopsis (Iwasaki *et al.* 2013; Machida *et al.* 2015). *rpn2a*, *rpn1a*, and *rpn8a* mutants all enhance the abaxialization phenotypes observed in *as2* mutants, demonstrating a requirement for 26SP function in the specification of organ polarity (Huang *et al.* 2006). Establishment of abaxial identify also requires *FIL*, another gene we have shown interacts genetically with BOB1. Supporting this connection among *BOB1* and polarity genes is the observation that the establishment of leaf polarity is sensitive to high temperatures. *as1* and *as2* mutants produce leaves with disrupted adaxial-abaxial polarity and one class of these leaves, called lotus leaves, occurs more frequently at high temperatures in an *er* (*i.e.*, Ler) background (Xu *et al.* 2003; Qi *et al.* 2004). There is significant overlap between the machinery required for maintaining proteostasis under normal conditions and the machinery required for responding to heat stress (Albanèse *et al.* 2006). This suggests that, at high temperatures, the machinery required for developmental proteostasis could be titrated away by stress induced misfolded proteins, uncovering phenotypes in cellular pathways such as the *AS1-AS2* pathway.

Plant hormones are important regulators of plant development and the ubiquitin-26SP system is integral to most plant hormone signal transduction pathways. In response to the presence of hormones, negative regulators are degraded by the 26SP, enabling rapid signal transduction (Santner and Estelle 2010). Our results suggest that, in addition to these very specific roles, the 26SP may also be required for ensuring normal development more generally by maintaining proteostasis in concert with protein chaperones.

The limitation of this work is that all of the interactions we have discovered are genetic. They do not provide direct mechanistic insights into the developmental requirements for *BOB1* activity. BOB1 may have undiscovered functions in addition to chaperone activity and it is conceivable that the *in vitro* assays used to demonstrate this activity do not accurately reflect BOB1’s cellular functions. The discovery of new *BOB1* functions could change our interpretation of our interaction data. Even without a full understanding of the mechanisms that underlie the genetic interactions between *BOB1* and the 26SP, their possible importance is highlighted by their conservation between plants and animals. An integrated *C. elegans* physical and genetic interaction network identified interactions supported by phenotypic and expression correlation data between *nud-1*, the *BOB1* homolog, and multiple proteasome subunits including *rpt-2*, *rpn-1*, and *rpn-8* (Gunsalus *et al.* 2005).

AS1 and AS2 form a heterodimeric complex that directly binds to the promoters of target genes (Guo *et al.* 2008). One explanation for our results is that this physical interaction requires *BOB1* and 26SP mediated proteostasis. In the absence of this quality control mechanism the AS1-AS2 complex would not function normally. FRET and yeast 2-hybrid approaches have been used to assay the AS1-AS2 interaction making it possible to test this hypothesis directly (Xu *et al.* 2003; Rast and Simon 2012).

## Supplementary Material

Supplemental Material is available online at www.g3journal.org/lookup/suppl/doi:10.1534/g3.117.300496/-/DC1.
